# Mygalomorph Spider Community of a Natural Reserve in a Hilly System in Central Argentina

**DOI:** 10.1673/031.012.3101

**Published:** 2012-03-01

**Authors:** Nelson Ferretti, Gabriel Pompozzi, Sofia Copperi, Fernando Pérez-Miles, Alda González

**Affiliations:** ^1^ Centro de Estudios Parasitológies y de Vectores CEPAVE (CCT- CONICET- La Plata) (UNLP), Calle 2 N° 584, (1900) La Plata, Argentina; ^2^ Departamento de Biología, Bioquímica y Farmacia, Universidad Nacional del Sur, Bahía Blanca, Buenos Aires, Argentina; ^3^ Facultad de Ciencias, Sección Entomología, Universidad de la República, Montevideo, Uruguay

**Keywords:** Araneae, diversity, ecology, Mygalomorphae, natural reserve

## Abstract

The diversity, abundance, spatial distribution, and phenology of the mygalomorph spider community in the “Ernesto Tornquist” Strict Nature Reserve were analyzed in this study. Located in southwestern Buenos Aires, Argentina, the Reserve is representative of the Ventania system, which is a sigmoidal mountain belt 180 km in length. This exceptional hilly ecosystem is home for many endemic species and rich native fauna and flora. Spider abundance was sampled monthly from October 2009 to October 2010 by hand capture and pitfall traps on grassland slopes. The species recorded in the study area were: *Actinopus* sp.1 (Actinopodidae); *Grammostola vachoni* and *Plesiopelma longisternale* (Theraphosidae); *Acanthogonatus centralis* (Nemesiidae); and *Mecicobothrium thorelli* (Mecicobothriidae). *Grammostola vachoni* and *Acanthogonatus centralis* were the dominant species in hand capture and pitfall traps, respectively. The seasonal variation, diversity, and abundance of the mygalomorph community are analyzed and discussed here. The Mygalomorphae of the Ventania system comprises an important group of sedentary and cryptozoic spiders that seem to be highly dependent on habitat type and environmental factors.

## Introduction

There have been many spider community studies in the neotropics (Candiani et al. 2005; Dias et al. 2005; Indicatti et al. 2005; Raizer et al. 2005; Sandoval 2005; Pinkus-Rendón et al. 2006; Podgaiski et al. 2007). As a typical megadiverse group, spiders have gained wide acceptance in ecological studies as indicators of environmental quality (Clausen 1986; Maelfait et al. 1990; Churchill 1997). In Argentina, research on ecological aspects of communities of spiders associated with natural (Corronca and Abdala 1994; Ávalos et al. 2005; Grismado 2007; Rubio et al. 2008; Ferretti et al. 2010a) and altered areas (Beltramo et al. 2006; Ávalos et al. 2007; Armendano and Gonzalez 2010) has been conducted, but knowledge gaps still exist for most of the natural areas. Moreover, there is no data on ecological aspects of the spider community in the Ventania system. Mygalomorph spiders are distributed worldwide and are well represented in the Neotropical region, although their ecology and natural history have mainly been studied in the Neartic (Baerg 1958; Minch 1979; Coyle and O'Shear 1981) and Australian regions (Main 1987; Jackson and Pollard 1990; Kotzman 1990). Studies regarding ecological aspects of mygalomorphs in the Neotropical region were done by Pérez-Miles et al. (1993) and Ferretti et al. (2010a). Many species of Mygalomorphae have long life cycles, living for 15–30 years and requiring 5– 6 years to reach reproductive maturity (Main 1978). Moreover, they are habitat specialists and females are sedentary (Main 1987; Coyle and Icenogle 1994), promoting geographic fragmentation over space and time and small geographic distributions (Bond et al. 2006).

The “Ernesto Tornquist” Strict Nature Reserve is located in the Ventania system and was created in 1937 to preserve this unique upland ecosystem, which contains native fauna and flora including many endemic species. From an ecological point of view, the Natural Reserve in this hilly system is one of the last relicts of more or less well—conserved areas in the Pampas ecoregion where several endemic taxa and habitat types can be found (Zalba and Cozzani 2004). The Ventania system is at the limit of the two phytogeographic provinces of Pampa and Espinal, and is home to more than 400 native plant species (Kristensen and Frangi 1995a), many of which are endemic and face extinction risks (i.e., *Polygala ventanensis* and *Senecio Leucopeplus*) (Villamil et al. 1996; Delucchi 2006). Because it is a protected area, it is imperative to know the biological diversity being preserved. Although there have been many studies on faunal diversity and conservation in the Ventania system, most were conducted on vertebrates (Cozzani et al. 2004, 2007; Di Giácomo 2005; Doiny Cabré and Lejarraga 2007; Cozzani and Zalba 2009) and insects (Konopko et al. 2009).

**Figure 1.**
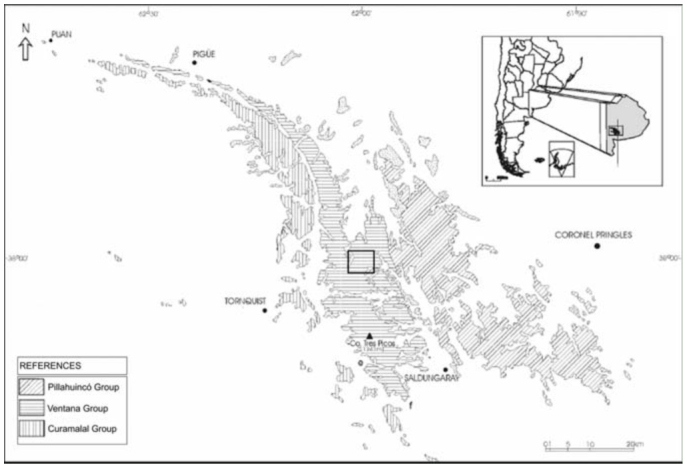
Geologic map of Ventania (modified from Suero 1972) showing the location of the “Ernesto Tornquist” Natural Reserve, where the study was carried out. High quality figures are available online.

The Ventania system is a hilly environment located in southwestern Buenos Aires, Argentina. It includes a 180 km long × 50 km wide mountain belt running northwest to southwest, and is composed of basement and sedimentary cover that can be divided into three groups: the Curamalal, Ventana, and Pillahuincó (Figure 1). Deformational episodes occurred during the Upper Devonian and Permian (Sellés-Martínez 2001; Gregory et al. 2005). Although the specifics of the development of this mountain range remain in controversy, the similarities between the surfaces and weathering products of the Buenos Aires ranges and the corresponding features of Cape Province in South Africa suggest a common Gondwanic origin for both landscapes (Keidel 1916; Du Toit 1927). The mountains that form the Ventania range culminate at varying altitudes and correspond to differentially uplifted blocks. Undulating between 800 and 900 m a.s.l. in midrange, it rises by 150 m in the southern part of the Sierra de la Ventana, where it is dominated by a few summits of up to 1240 m a.s.l., and descends to approximately 700 m in the north (Demoulin et al. 2005). The purpose of this paper is to assess the diversity, abundance, spatial distribution, and phenology of a mygalomorph spider community at the “Ernersto Tornquist” Strict Nature Reserve.

## Materials and Methods

### Study area

**Figure 2.**
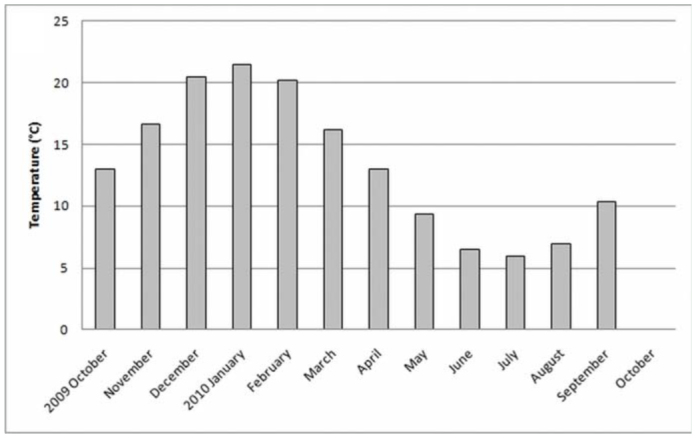
Average monthly temperature of the “Ernesto Tornquist” Natural Reserve. High quality figures are available online.

**Figure 3.**
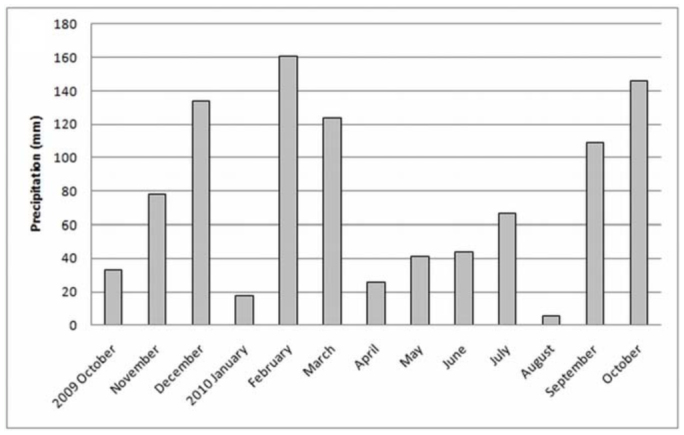
Rainfall amounts at the “Ernesto Tornquist” Natural Reserve. High quality figures are available online.

The study area is located in the Ventania system in southwestern Buenos Aires, Argentina, at an elevation of 650 m above sea level. The “Ernesto Tornquist” Strict Nature Reserve (38° 00'-38° 07' S and 61°52'-62° 03' W) is located inside this hilly system (Figure 1), and has an area of approximately 6700 ha. The topography ranges from steep slopes at high elevations of the mountain system to gentler slopes at lower levels (piedmont). The climate is humid and temperate with an average annual rainfall of 850 mm that decreases from NE to SW during fall and spring. Rainfall increases with altitude, from 745 mm at the lowest altitude to 828 mm at peaks (Pérez and Frangi 2000). The mean annual temperature is 14.5 °C and similarly decreases from northeast to south. An altitudinal gradient of temperature is evident inside this hilly system, showing a decrease of 6.9 °C per 1000 m (Kristensen and Frangi 1995b). The natural vegetation consists of more than 400 native species with high endemism. On grassland slopes, species such *Briza subaristata*, *Stipa ambigua*, *S. caudata*, and *S. neesiana* are common. *Paspalum quadrifarium* covers the humid slopes, and endemic gramineous species such as *Festuca ventanicola*, *F. pampeana*, and *Stipa pampeana* are present above 500 m a.s.l. (Frangi and Bottino 1995). The average monthly temperature and rainfall changes during the study period (Figures 2 and 3) were obtained from a station located at the base of the hill “Cerro Bahía Blanca” at 2 km from the study site. Temperature and precipitation measurements were recorded daily and compiled into monthly totals.

### Spider sampling and identification

**Figure 4.**
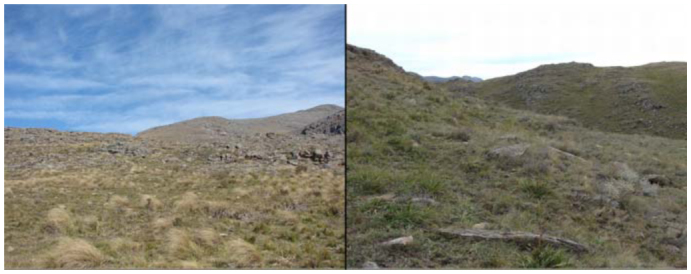
Typical grassland slopes in the “Ernesto Tornquist” Natural Reserve where the array of pitfall traps was located. High quality figures are available online.

Samples were taken monthly from October 2009 to October 2010. Two techniques were used: hand capture and pitfall trapping. Traps were arranged in a line of 10 placed each 10 m along a transect of 100 m parallel to the longest axis of a grassland slope with native vegetation (Figure 4). Pitfall traps consisted of cylindrical plastic containers 23 cm in diameter and 15 cm in height buried and covered with a plastic roof supported by three metallic rods 15 cm above the soil. They were filled with 1500 mL of ethylene glycol, which prevented evaporation and acted as a preservative. All traps were examined every 30 days and were refilled.

Spiders were hand collected by searching in potential cryptozoic refuges such as under rocks, logs, and dung. Spiders were collected in successive and adjacent transects involving three strips measuring 250 m long and 3 m wide for each collector each month. These strips were 100 m away from the pitfall line and were displaced 100 m from each other. Hand collecting involved three collectors, and each spending approximately four hours in each plot. The sampling area included approximately 0.5 ha of native grassland slopes.

Spiders were separated from debris, washed, and stored in 70% ethanol. Specimens were identified at the species level following Holmberg (1882), Schiapelli and Gerschman (1960, 1970), Goloboff (1995), Raven (1985), Ferretti et al. (2010b). *Actinopus* sp.1 constituted the only morphospecies that could not be identified at the species level. Voucher specimens will be deposited in the Museo de La Plata, División Entomología in Argentina.

### Statistical analyses

Normality and homogeneity of variances were evaluated with Levene and Shapiro-Wilk tests. Analysis of variance (ANOVA) tests were made to compare the abundances of spiders between sampling seasons. Pearson correlation was used to explore possible linear relationships of abundance with temperature and precipitation. All statistical analyses were performed using PAST version 1.89 (Hammer et al. 2001). To determine the effect of sample abundance on sample richness, the rarefaction was made using EstimateS v8.0 (Colwell 2006) based on the number of individuals (Gotelli and Colwell 2001).

## Results

### Taxonomic composition, species richness and demographic structure

**Figure 5.**
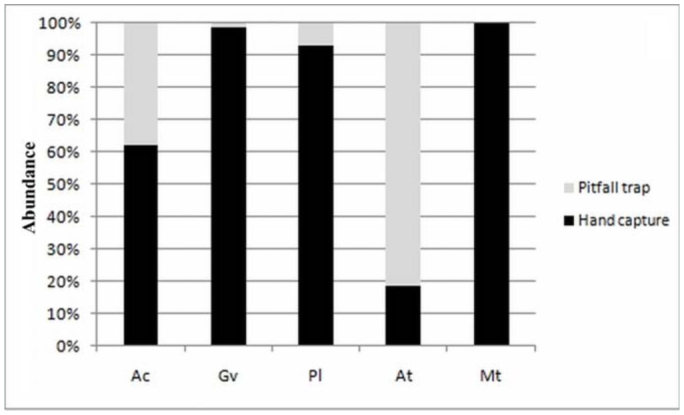
Relative abundances of mygalomorph spiders collected by hand capture and pitfall traps in “Ernesto Tornquist” Natural Reserve. **Ac**, *Acanthogonatus centralis*; **Gv**, *Grammostola vachoni*; **Pl**, *Plesiopelma longisternale*; **At**, *Actinopus* sp.1; **Mt**, *Mecicobothrium thorelli.* High quality figures are available online.

**Figure 6.**
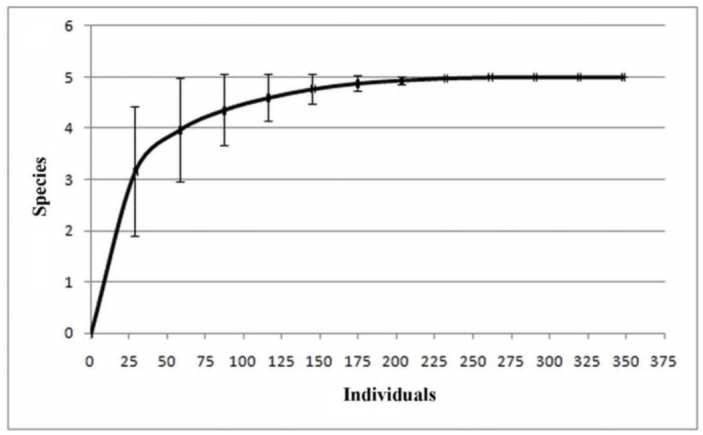
Rarefaction curve of hand capture and pitfall traps in “Ernesto Tornquist” Natural Reserve based on number of individual of mygalomorph spiders. High quality figures are available online.

In total, 426 individuals of Mygalomorphae were collected during the sampling period: 349 were collected by hand capture and 77 by pitfall traps (these values do not include juveniles found with mothers). The species recorded in the study area belong to four families: *Actinopus* sp.1 (Actinopodidae); *Grammostola vachoni* Schiapelli and Gerschman 1960 and *Plesiopelma longisternale* (Schiapelli and Gerschman 1942) (Theraphosidae); *Acanthogonatus centralis* Goloboff 1995 (Nemesiidae); and *Mecicobothrium thorelli* Holmberg 1882 (Mecicobothriidae). The absolute and relative frequencies of individuals collected by hand capture and with pitfall traps are shown in Table 1. *Grammostola vachoni* clearly was the dominant species in hand capture, constituting more than 50% of collected individuals. In pitfall traps, the dominant species was *A. centralis*, representing 75.32% of individuals recorded (Table 1).The number of species achieved by the sample techniques was five species and four species with hand capture and pitfall traps, respectively; *M. thorelli* was not found in any traps. Hand capture was more efficient for the theraphosids *G. vachoni* and *P. longisternale*, and was clearly effective for *M. thorelli* (Mecicobothriidae) (Figure 5). Both techniques were effective to capture *A. centralis* (Nemesiidae), and pitfall traps were more efficient for *Actinopus* sp.1 (Actinopodidae) (Figure 5).The rarefaction curve of hand captures and pitfall traps based on individual number (Figure 6) showed that more than 50% of species were recorded after collecting approximately 30 individuals. The total number of species was achieved after collecting approximately 200 individuals.

**Table 1.**
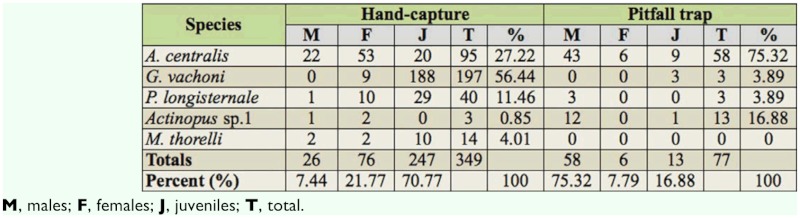
Absolute and relative frequencies of mygalomorph spiders collected by hand capture and pitfall traps in “Ernesto Tornquist” Natural Reserve.

With hand capture, juveniles clearly prevailed over adults, with a 70.77% of the individuals collected. However, in pitfall traps, adults (83.1%) prevailed over juveniles. Males were most frequent in pitfall traps, representing 75.32% of the total, while females and juveniles were less frequent at 7.79% and 16.88%, respectively.

### Seasonal variation

**Figure 7.**
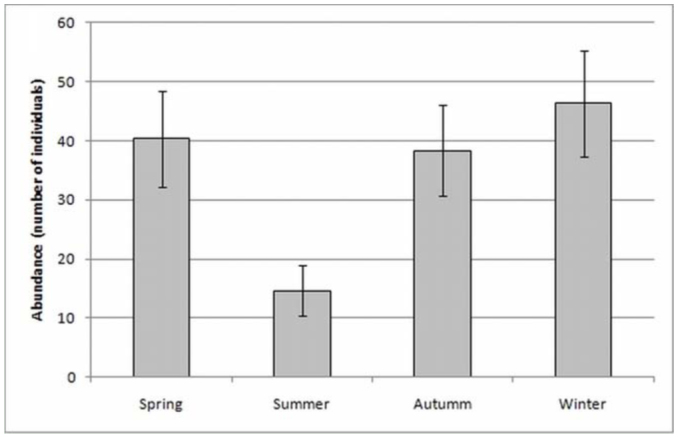
Seasonal abundance of mygalomorph spiders collected. High quality figures are available online.

**Figure 8.**
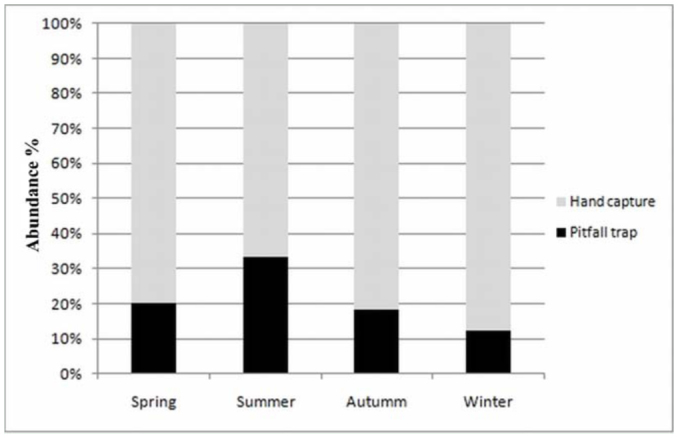
Seasonal abundance of mygalomorph spiders collected by hand capture and pitfall traps in “Ernesto Tornquist” Natural Reserve. High quality figures are available online.

The seasonal analysis of hand captures and pitfall trap samples showed approximately the same abundances of spiders for spring (September, October, and November), fall (March, April, and May), and winter (June, July, and August), and a lower abundance during summer in the Southern Hemisphere (December, January, and February) (Figure 7). However, no significant differences of abundances were found between seasons (ANOVA, *F* = 3.44, *p* > 0.05). The efficiency of sampling techniques was similar in spring, fall, and winter, but in the summer the abundance of individuals recorded in pitfall traps was higher (Figure 8), although summer was the less abundant season.

**Figure 9.**
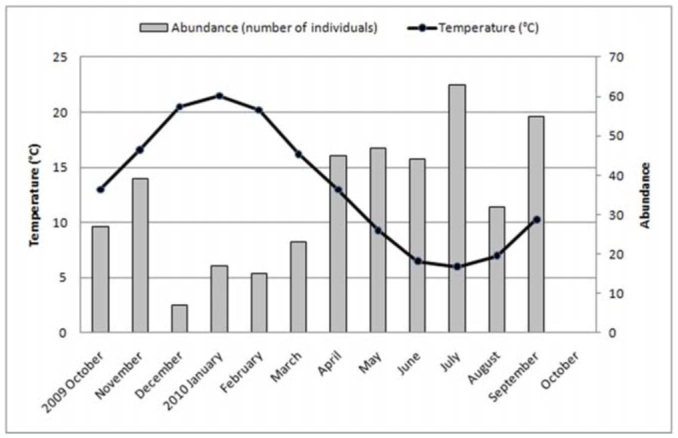
Monthly abundance of mygalomorph spiders with the monthly average temperature in the “Ernesto Tornquist” Natural Reserve. High quality figures are available online.

**Figure 10.**
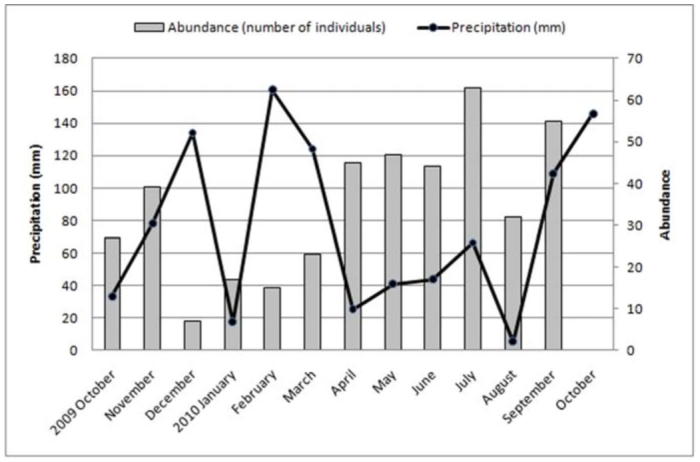
Monthly abundance of spiders with the rainfall amounts in the “Ernesto Tornquist” Natural Reserve during the sampling period. High quality figures are available online.

The highest values of abundances of Mygalomorphae corresponded with lower values of temperatures in the study area during the sampling period, and conversely, the lower values of abundances were recorded in summer (December–February), corresponding with the highest values of temperature in the area (Figure 9) (r = —0.810, *p* < 0.01). Regarding the values of precipitation during the sampling period in the area, no correlation was found with abundance of mygalomorph spiders (r = —0.325, *p* = 0.302) (Figure 10). However, in the summer and fall, an increase in precipitation above 100 mm clearly diminished the abundance of Mygalomorphae in the study area.

### Phenology

**Figure 11.**
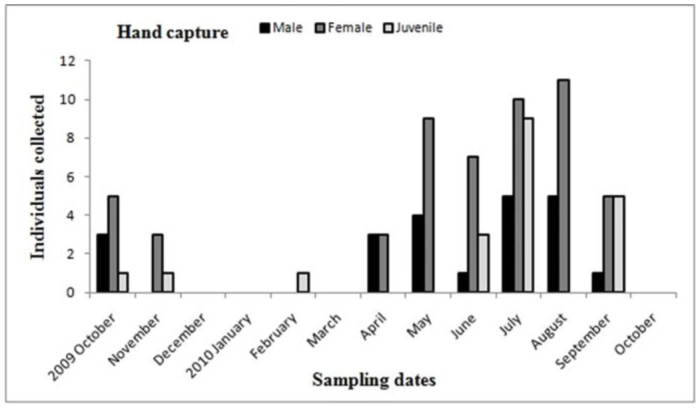
*Acanthogonatus centralis.* Phenology based on specimen activity (individuals/month) in “Ernesto Tornquist” Natural Reserve by hand capture. High quality figures are available online.

**Figure 12.**
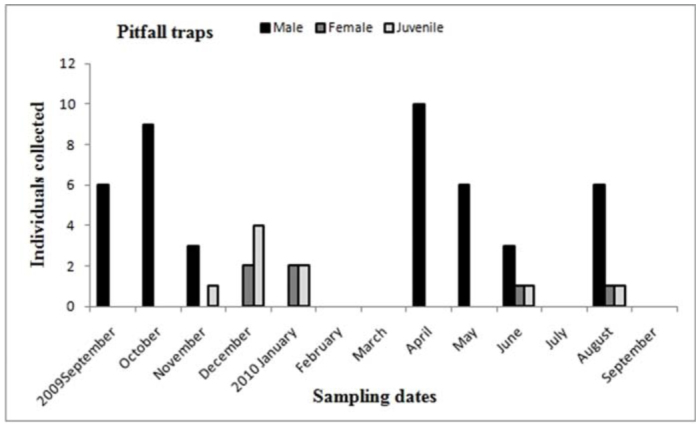
*Acanthogonatus centralis.* Phenology based on specimen activity (individuals/month) in “Ernesto Tornquist” Natural Reserve using pitfall traps. High quality figures are available online.

**Figure 13.**
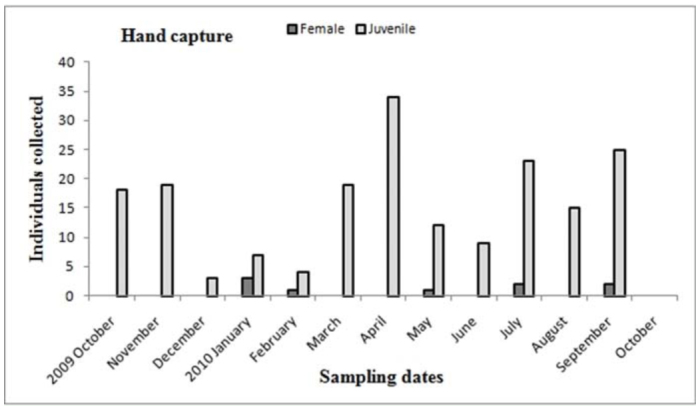
*Grammostola vachoni.* Phenology based on specimen activity (individuals/month) in “Ernesto Tornquist” Natural Reserve by hand capture. High quality figures are available online.

**Figure 14.**
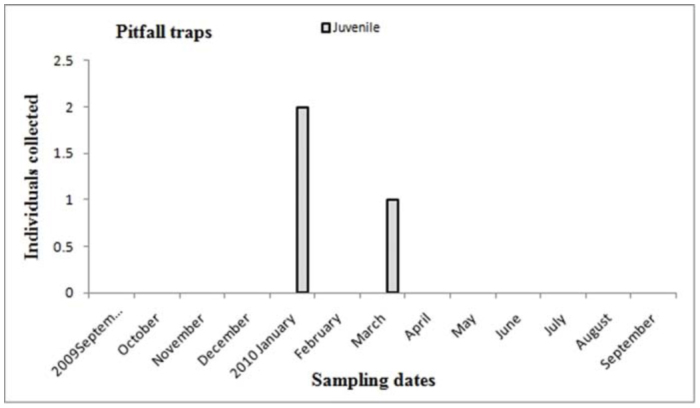
*Grammostola vachoni.* Phenology based on specimen activity (individuals/month) in “Ernesto Tornquist” Natural Reserve using pitfall traps. High quality figures are available online.

**Figure 15.**
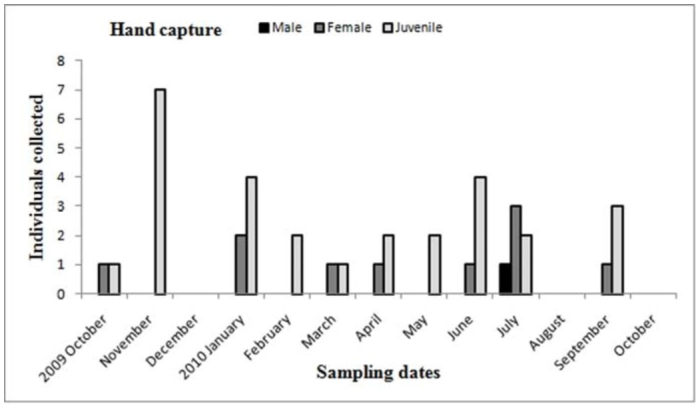
*Plesiopelma longisternale.* Phenology based on specimen activity (individuals/month) in “Ernesto Tornquist” Natural Reserve by hand capture. High quality figures are available online.

**Figure 16.**
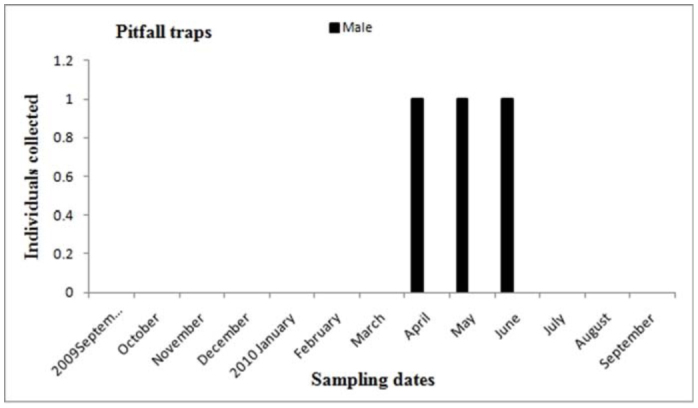
*Plesiopelma longisternale.* Phenology based on specimen activity (individuals/month) in “Ernesto Tornquist” Natural Reserve using pitfall traps. High quality figures are available online.

**Figure 17.**
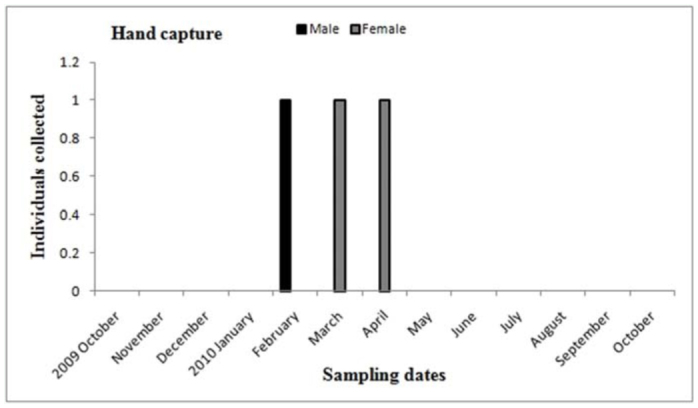
*Actinopus* sp.1. Phenology based on specimen activity (individuals/month) in “Ernesto Tornquist” Natural Reserve. Hand captured. High quality figures are available online.

**Figure 18.**
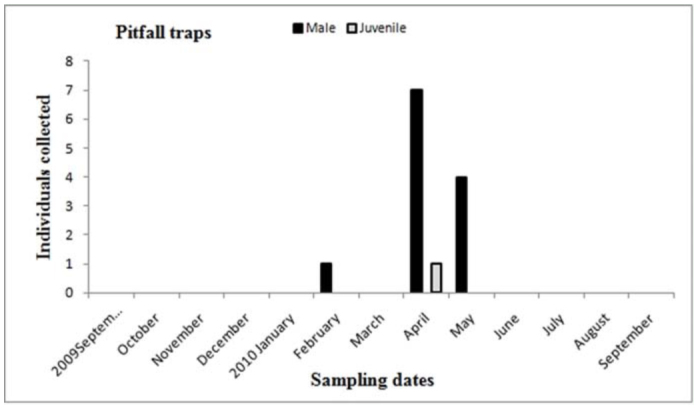
*Actinopus* sp.1. Phenology based on specimen activity (individuals/month) in “Ernesto Tornquist” Natural Reserve captured in pitfall traps. High quality figures are available online.

Males of *A. centralis* were recorded from April to November, corresponding to the end of fall, winter, and spring in the Southern Hemisphere (Figures 11 and 12). Although males of *A. centralis* seemed to be present during the entire sampling period, two activity periods were recorded in pitfall traps: one in April, May, and June (fall and beginning of winter), and the other in August, September, and October (end of winter and into spring). Males were recorded during months of medium and low temperatures (Figure 2) and low and high values of precipitation (Figure 3). Females and juveniles were abundant during the entire sampling period (Figures 11 and 12) excluding March. Juveniles showed higher abundance than males and females in pitfall traps during summer (January and February) (Figure 12). No males of *G. vachoni* were found by either hand capture or pitfall trap. Females were observed in summer (January and February) and also in May, July, and September (Figure 13). No females were captured in pitfall traps. One female of *G. vachoni* was found holding an egg sac during January. Juveniles were observed during the entire sampling period with a clearly active period in summer and the beginning of fall (Figures 13 and 14).The highest activity for males of *P. longisternale* was observed from April to June (fall and beginning of winter) (Figures 15 and 16), corresponding with the lower values of temperature (Figure 2) and precipitation (Figure 3) in the study area. Females were more abundant than males but were recorded in almost the entire sampling period. Two females of *P. longisternale* were found with egg sacs during January. Juveniles were more abundant than males and females, with the highest activity in November (end of spring) (Figure 15). Males of *Actinopus* sp.1 were clearly present during April and May (fall) (Figures 17 and 18). This activity corresponded to medium temperatures (Figure 2) and low precipitation (Figure 3) in the study area. Also, this species was captured in February, March, and April (Figure 17), but at a lower abundance. In this period, the values of both temperature (Figure 2) and precipitation were higher in the area during this sampling period (Figure 3). Females were less abundant and were observed in fall (Figure 17). In March, one female was found in a burrow with 12 juveniles that had recently emerged from the egg sac.

**Figure 19.**
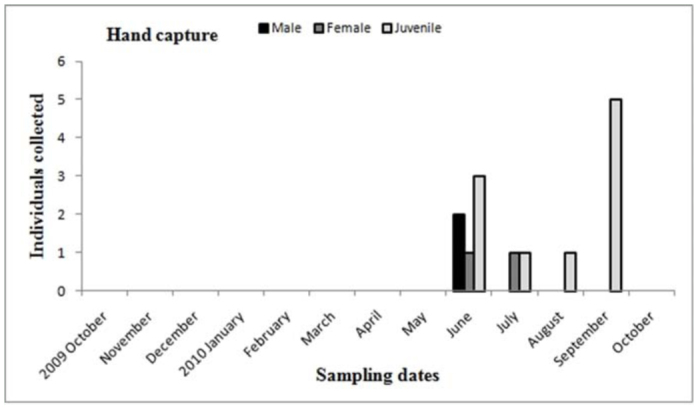
*Mecicobothrium thorelli.* Phenology based on specimen activity (individuals/month) in “Ernesto Tornquist” Natural Reserve. High quality figures are available online.

*Mecicobothrium thorelli* was collected from June to September (winter and beginning of spring) (Figure 19). Males showed one clear activity period in June corresponding with low temperature (Figure 2) and precipitation in the study area. Females were recorded in June and July. Juveniles were recorded from June to September (Figure 19) (September showed higher value of precipitation) (Figure 3).

## Discussion

The species richness recorded in the study area was similar to that found in other areas from Argentina (Ávalos et al. 2005; Ferretti et al. 2010a), and also from other Neotropical areas such as Amazonia (Höfer 1990), Bolivia (Sandoval 2005), and Uruguay (Pérez-Miles et al. 1993), ranging from four to six mygalomorph species. The number of species recorded in the study area was underestimated due to the absence of the species *Calathotarsus simoni* (Migidae), cited on Sierra de la Ventana and Tandil. This species is a trap—door spider, with scarce records in Museum collections. The only contributions about *C. simoni* (Schiapelli and Gerschman 1973, 1975) explained that specimens were found on a hilly slope where they construct their trap—doors covered with moss and ferns, making them extremely difficult to be located by collectors. It is possible that *C. simoni* may be absent in the study area; however, one male and many females were previously collected approximately 3 km from this area (Schiapelli and Gerschman 1975).

Obtaining 50% of the species with a low number of samplings, and the achievement of the total number of species by adding a few sampling dates in both techniques, indicates a relatively good sampling for the studied area despite the absence of *C. simoni.* The high abundances of *G. vachoni* and *P. longisternale* found via hand collection are explained by the greater efficacy of this method for finding species that live under stones, fallen trees, and dung; these species do not fall into pitfall traps, requiring more active searching (Pérez-Miles et al. 1993; Ferretti et al. 2010a). The juveniles of these theraphosid species prevailed over the adults in hand capture. This proportion probably reflects the extended juvenile stage, or the longevity of females and the short lifespan of adult males (Pérez-Miles et al. 1993; Costa and PérezMiles 2002; Ferretti et al. 2010a), as well as their cryptic habitat during this stage and small expansion range around burrows (PérezMiles et al. 1993; Shillington and McEwen 2006). The low abundances of theraphosid species in pitfall traps also could be explained by the presence of adherent organs on tarsi (claw tufts and scopulate) and their “cautious” locomotion (Pérez-Miles et al. 1993).

In the nemesiid *A. centralis*, the similar captures by hand and traps is probably explained by high individual motility. A high motility of the nemesiid species, *Stenoterommata platensis*, was observed on Martin Garcia Island (Argentina) (Ferretti et al. 2010a). The trap—door spider *Actinopus* sp. 1 also showed high captures in pitfall traps, but involved only wandering males. This is typical for trap—door species that are difficult to collect by hand capture (Pérez-Miles et al. 1993; Indicatti et al. 2005; Ferretti et al. 2010a). *Mecicobothrium thorelli*, also a cryptic species, was collected only by hand capture and appears to be a species with low motility. Males and juveniles of *M. thorelli* were recorded in pitfall traps next to a stream in a typical hill of Uruguay (Pérez-Miles et al. 1993). This species occupies habitats with high values of humidity (Costa and PérezMiles 1998). Moreover, in Uruguay, *M. thorelli* was found under stones, roots, and trunks in hilly areas of a streamside forest, as these spiders are highly sensitive to humidity variations (Costa and Pérez-Miles 1998). Pitfall traps in our study were not placed next to a stream, thus explaining the possible absence of this species by this method. However, *M. thorelli* was found occupying large stones on a grassland slope, but not in proximity to streams, appearing to be more tolerant to drier habitats in this study area.

Although no significant differences were found in mygalomorph abundances between seasons, summer was the period with lower abundance. This could be due to high values of temperature and precipitation (above 100 mm) in the study area creating unstable conditions, alternating between dry and humid periods. Moreover, the high proportion of individuals captured in pitfall traps occurred in the summer (more than 30%), which could be due to greater transit of individuals in relation with more prey availability in the area (Uetz 1976; Riechert and Luczark 1982).

## Phenology

The presence of walking males of mygalomorph spiders comprises an indicator of the mating period (Pérez-Miles et al. 1993; Ferretti et al. 2010a). The highest activity periods for males of *A. centralis* were recorded from the end of fall, winter, and spring, having two clear activity peaks. Other nemesiids, such *Stenoterommata* spp. in Uruguay and Brazil, also showed sexual activity peaks in the fall and spring (PérezMiles et al. 1993; Indicatti et al. 2008). However, *S. platensis* on Martin Garcia Island (Argentina) has their reproductive period in summer and fall (Ferretti et al. 2010a). Females were abundant in summer in pitfall traps, and via hand capture were abundant in fall, winter, and spring. The activity peak of females in the summer was also observed for *S. platensis* on Martin Garcia Island (Ferretti et al. 2010a). The presence of small juveniles in pitfall traps during summer (December and January) could indicate the emergence of juveniles and dispersion stages at this period (Pérez-Miles et al. 1993; Reichling 2000; Shillington and McEwen 2006).

Unfortunately, no males of *G. vachoni* were recorded on the study area. Females showed an even seasonal distribution with hand capture and were absent in pitfall traps. Juveniles were abundant in January (summer) and March (fall), indicating the emerging and dispersion stage as was found for other theraphosid species (Pérez-Miles et al. 1993; Ferretti et al. 2010a). Moreover, the presence of a female holding an egg sac in a warmer month (January) supported this hypothesis.

The other theraphosid species recorded in the study area, *P. longisternale*, showed one clear activity peak, with males being abundant in the fall and beginning of winter. This activity period could be the same for *P. longisternale* in Uruguay, where one male was recorded in May by hand capture (Pérez-Miles et al. 1993). These authors did not record males of *P. longisternale* in pitfall traps, perhaps because the traps they used were smaller than in our study (Pérez-Miles et al. 1993). Males of *P. longisternale* were recorded in pitfall traps using wider traps. The winter activity peak could be a mechanism to avoid potential predators that show low frequencies and activity during this period (Pérez-Miles et al. 1993; Ferretti et al. 2010a). Females and juveniles showed an even seasonal distribution and were absent in pitfall traps. The presence of females holding egg sacs during January suggested the same emergence and dispersion stage period as the other theraphosid recorded, *G. vachoni*; thus, a strong interspecific competition for resources such as burrows and prey are expected between juveniles of these species.

Males of *Actinopus* sp. showed two activity periods, with a period of higher abundance in the fall and lesser one in the summer. Sexual activity in the fall could be the same as that registered for other *Actinopus* spp. in Uruguay and on Martin Garcia Island (Argentina) (Pérez-Miles et al. 1993; Ferretti et al. 2010a). However, in our study area, two males were captured in summer (February). This may support the hypothesis of two different species, but a taxonomical review of the genus *Actinopus* in Argentina is needed. A female found with spiderlings in March and also other found with her offspring by Ferretti et al. (2010a) in spring (October) additionally supports the existence of two species of *Actinopus* in Buenos Aires province.

*Mecicobothrium thorelli* was the species with the more restricted activity period during the sampling, being abundant in winter but also found in early spring. Males were found exclusively in June (winter), one of the cooler months in the study area. Adult males were found from May to September, with a peak of activity in July (Pérez-Miles et al. 1993). These authors interpreted the winter activity as a means to avoid predation, and this could be operating in the same way for this species in the study area. Females were also abundant in June and July (winter), and juveniles showed an activity peak in September (beginning of spring), maybe the emergence period of spiderlings. Females of *M. thorelli* made egg sacs in August and September, and juveniles emerge after a month of the egg sac construction (Costa and Pérez-Miles 1998).

Overall, the Mygalomorphae of the Ventania system comprises an important group of sedentary and cryptozoic spiders that seem to be highly dependent on habitat type and environmental factors. The diversity and abundance of these spiders in the study area is higher in relation to other areas (Pérez-Miles et al. 1993; Ávalos et al. 2005; Ferretti et al. 2010a), and the microclimatic conditions (Kristensen and Frangi 1995a, 1995b) and vegetation (Lizzi et al. 2007) of the hilly system of Ventania could provide a suitable habitat for these cryptozoic species. The present study constitutes the first on the Mygalomorphae spider community in the Strict Nature Reserve “Ernesto Tornquist” in Buenos Aires, Argentina. Moreover, the knowledge of spider fauna on this Natural Reserve could help in the preservation of natural grassland habitats. This area is important, because to ensure the conservation of regional diversity, further studies on natural grassland habitats such as this one will prove necessary to inform management and conservation decisions.

## References

[bibr01] Armendano A, González A. (2010). Comunidad de aranas (Arachnida, Araneae) del cultivo de alfalfa (*Medicago sativa*) en Buenos Aires, Argentina.. *Revista de Biología Tropical*.

[bibr02] Ávalos G, Rubio GD, Bar ME, Damborsky MP, Oscherov EB (2005). Composición y distribución de la araneofauna del Iberá. Resúmenes de las Comunicaciones Científicas y Tecnológicas, Univ.. Nacional del Nordeste..

[bibr03] Ávalos G, Rubio GD, Bar ME, Gonzalez A. (2007). Aranas (Arachnida: Araneae) asociadas a dos bosques degradados del Chaco húmedo en Corrientes, Argentina.. *Revista de Biología Tropical*.

[bibr04] Baerg WJ. (1958). *The Tarantula.*.

[bibr05] Beltramo J, Bertolaccini I, González A. (2006). Spiders of soybean crops in Santa Féprovince, Argentina: Influence of surrounding spontaneous vegetation on lot colonization.. *Brazilian Journal of Biology*.

[bibr06] Bond JE, Beamer DA, Lamb T, Hedin M. (2006). Combining genetic and geospatial analyses to infer population extinction in mygalomorph spiders endemic to the Los Angeles region.. *Animal Conservation*.

[bibr07] Candiani DF, Indicatti RP, Brescovit AD. (2005). Composição e diversidae da araneofauna (Araneae) de Serapilheira em tres florestas urbanas na cidade de São Paulo, São Paulo, Brasil.. *Biota Neotropica*.

[bibr08] Churchill TB. (1997). Effects of sampling method on composition of a Tasmanian coastal heathland spider assemblages.. *Memoirs of the Queensland Museum*.

[bibr09] Clausen IH. (1986). The use of spiders (Araneae) as ecological indicators.. *Bulletin of the British Arachnological Society*.

[bibr10] Colwell RK (2006). EstimateS: Statistical estimation of species richness and shared species from samples..

[bibr11] Corronca JA, Abdala CS. (1994). La fauna araneológica de la Reserva Ecológica “E1 Bagual”, Formosa, Argentina.. *Aracnología*.

[bibr12] Costa FG, Pérez-Miles F. (1998). Behavior, life cycle, and webs of *Mecicobothrium* thorelli.. *Journal of Arachnology*.

[bibr13] Costa FG, Pérez-Miles F. (2002). Reproductive biology of Uruguayan theraphosids (Araneae, Theraphosidae).. *Journal of Arachnology*.

[bibr14] Coyle FA, O'Shear WA. (1981). Observations on the natural history of *Sphodros abboti* and *Sphodros rufipes* (Araneae, Atypidae), with evidence for a contact sex pheromone.. *Journal of Arachnology*.

[bibr15] Coyle FA, Icenogle WR. (1994). Natural history of the California trapdoor spider genus *Aliatypus* (Araneae, Antrodiaetidae).. *Journal of Arachnology*.

[bibr16] Cozzani N, Zalba S. (2009). Estructura de la vegetación y selección de hábitats reproductivos en aves del pastizal pampeano.. *Ecología Austral*.

[bibr17] Cozzani N, Sánchez R, Zalba SM. (2004). Nidificación de la loica pampeana (*Sturnella defilippii*) en la provincia de Buenos Aires.. *Hornero*.

[bibr18] Cozzani N, Zalba SM, Mattos E, Sarria R. (2007). Nidificación del Jilguero Austral (*Sicalis lebruni*) en Sierra de la Ventana, provincia de Buenos Aires.. *Nuestras Aves*.

[bibr19] Delucchi G. (2006). Las especies vegetales amenazadas de la provincia de Buenos Aires: Una actualización.. *APRONA*.

[bibr20] Demoulin A, Zarate M, Rabassa J. (2005). Long—term landscape development: a perspective from the southern Buenos Aires ranges of east central Argentina.. *Journal of South American Earth Science*.

[bibr21] Dias MF, Brescovit AD, Menezes M. (2005). Aranhas de solo (Arachnida: Araneae) em diferentes fragmentos florestais no sul da Bahia, Brasil.. *Biota Neotropica*.

[bibr22] Di Giacomo AS. (2005). Areas Importantes para la Conservación de las Aves en Argentina. Sitios prioritarios para la conservación de la biodiversidad. Temas de naturaleza y conservación.. *Aves argentinas*.

[bibr23] Doiny Cabré PC, Lejarraga L. (2007). *Aves de Sierra de la Ventana.* Published by the author..

[bibr24] Du Toit AL. (1927). *A Geological Comparison of South America with South Africa.*.

[bibr25] Ferretti N, Pérez-Miles F, González A. (2010a). Mygalomorph spiders of the Natural and Historical Reserve of Martin Garcia Island, Rio de la Plata River, Argentina.. *Zoological Studies*.

[bibr26] Ferretti N, Pompozzi G, Copperi S, González A, Pérez-Miles F. (2010b). Arañas Mygalomorphae de la provincia de Buenos Aires, Argentina: clave para la determinación de especies.. *Bioscriba*.

[bibr27] Frangi JL, Bottino OJ. (1995). Comunidades vegetales de la Sierra de la Ventana, Provincia de Buenos Aires.. *Revista de la Facultad de Agronomía* (*La Plata*).

[bibr28] Goloboff PA. (1995). A revision of the South American spiders of the family Nemesiidae (Araneae, Mygalomorphae). Part I: Species from Peru, Chile, Argentina and Uruguay.. *Bulletin of the American Museum of Natural History*.

[bibr29] Gotelli NJ, Colwell RK. (2001). Quantifying biodiversity: procedures and pitfalls in the measurement and comparison of species richness.. *Ecology Letters*.

[bibr30] Gregory DA, López VL, Grecco LE. (2005). A Late Proterozoic—Early Paleozoic magmatic cycle in Sierra de la Ventana, Argentina.. *Journal of South American Earth Science*.

[bibr31] Grismado C. (2007). Comunidades de aranas de la Réserva Natural Otamendi, Provincia de Buenos Aires. Riqueza específica y Diversidad..

[bibr32] Hammer O, Harper DAT, Ryan PD. (2001). PAST: Paleontological Statistics software package for education and data analysis..

[bibr33] Höfer H. (1990). The spider community (Araneae) of a Central Amazonian blackwater inundation forest (Igapó).. *Acta Zoologica Fennica*.

[bibr34] Holmberg E. (1882). Observations a propos de sous-ordre des araingnées territelaires (Territelaire).. *Boletín Académico Argentino*.

[bibr35] Indicatti RP, Candiani DF, Brescovit AD, Japyassú HF. (2005). Diversidade de aranhas (Arachnida, Araneae) de solo na bacia do reservatório do Guarapiranga, São Paulo, São Paulo, Brasil.. *Biota Neotropica*.

[bibr36] Indicatti RP, Lucas SM, Ott R, Brescovit AD. (2008). Litter dwelling mygalomorph spiders (Araneae: Microstigmatidae, Nemesiidae) from Araucaria Forests in southern Brazil, with the description of five new species.. *Revista Brasileira de Zoologia*.

[bibr37] Jackson RR, Pollard SD. (1990). Intraspecific interactions and the function of courtship in mygalomorph spiders: a study of *Porrothele antipodiana* (Araneae, Hexathelidae) and a literature review.. *New Zealand Journal of Zoology*.

[bibr38] Kotzman M. (1990). Annual activity patterns of the Australian tarantula *Selenoscomia stirlingi* (Araneae, Theraphosidae) in an arid area.. *Journal of Arachnology*.

[bibr39] Keidel J. (1916). La geología de las sierras de la Provincia de Buenos Aires y sus relaciones con las montañas de Sud-África y los Andes. *Anales del Ministerio de Agricultura de la Nación.*. *Geología*, *Mineralogía y Minería*.

[bibr40] Konopko SA, Mazzuconni SA, López Ruf ML, Bachman AO. (2009). Los heterópteros acuáticos y semiacuáticos del Parque Provincial Ernesto Tornquist (Provincia de Buenos Aires, República Argentina).. *Revista de la Sociedad Entomológica Argentina*.

[bibr41] Kristensen MJ, Frangi JL. (1995a). La Sierra de La Ventana: una isla de biodiversidad.. *Ciencia Hoy*.

[bibr42] Kristensen MJ, Frangi JL. (1995b). Mesoclimas de pastizales de la Sierra de la Ventana.. *Ecología Austral*.

[bibr43] Lizzi JM, Garbulsky MF, Golluscio RA, Deregibus AV. (2007). Mapeo indirecto de la vegetación de Sierra de la Ventana, provincia de Buenos Aires.. *Ecología Austral*.

[bibr44] Maelfait J, Jocque R, Baert L, Descender K. (1990). Heathland management and spiders.. *Acta Zoologica Fennica*.

[bibr45] Main BY. (1978). Biology of the arid—adapted Australian trapdoor spider *Anidiops villosus* (rainbow).. *Bulletin of the British Arachnological Society*.

[bibr46] Main BY., Majer J (1987). Ecological disturbance and conservation of spiders: implications for biogeographic relics in southwestern Australia.. *The Role of Invertebrates in Conservation and Biological Surveys*.

[bibr47] Minch EW. (1979). Burrow entrance plugging behavior in the tarantula *Aphonopelma chalcodes* Chamberlin (Araneae: Theraphosidae).. *Bulletin of the British Arachnological Society*.

[bibr48] Pinkus-Rendón MA, León-Cortés JL, IbarraNúñez G. (2006). Spider diversity in a tropical habitat gradient in Chiapas, Mexico.. *Diversity and Distributions*.

[bibr49] Pérez CA, Frangi JL. (2000). Grassland biomass dynamics an altitudinal gradient in the Pampa.. *Journal of Range Management*.

[bibr50] Pérez-Miles F, Costa FG, Gudynas E. (1993). Ecología de una comunidad de Mygalomorphae criptozoicas de Sierra de las Animas, Uruguay (Arachnida, Araneae).. *Aracnología*.

[bibr51] Podgaiski LR, Ott R, Lopes-Rodriguez EN, Buckup EH, Marques MA. (2007). Araneofauna (Arachnida; Araneae) do Parque Estadual do Turvo, Rio Grande do Sul, Brasil.. *Biota Neotropica*.

[bibr52] Raizer J, Japyassú HF, Indicatti RP, Brescovit AD. (2005). Comunidade de aranhas (Arachnida, Araneae) do pantanal norte (Mato Grosso, Brasil) e sua similaridade com a araneofauna amazónica.. *Biota Neotropica*.

[bibr53] Raven RJ. (1985). The spider infraorder Mygalomorphae (Araneae): cladistics and systematics.. *Bulletin of the American Museum of Natural History*.

[bibr54] Reichling SB. (2000). Group dispersal in juvenile *Brachypelma vagans* (Araneae, Theraphosidae).. *Journal of Arachnology*.

[bibr55] Riechert SE, Luczak J., Witt PN, Rovner JS (1982). Spider foraging: behavioral responses to prey.. *Spider Communication. Mechanisms and Ecological Significance*.

[bibr56] Rubio GD, Corronca JA, Damborsky MP. (2008). Do spider diversity and assemblages change in different contiguous habitats? A case study in the protected habitats of the Humid Chaco ecoregion, north—east Argentina.. *Environmental Entomology*.

[bibr57] Sandoval LC. (2005). Reporte sobre la riqueza de arañas (Araneae) en tres tipos de vegetación de la reserva municipal Valle de Tucavaca.. *Kempffiana*.

[bibr58] Schiapelli RD, Gerschman de Pikelin BS. (1942). Arañas argentinas (1° parte).. *Anales del Museo Argentino de Ciencias Naturales*, *Entomología*.

[bibr59] Schiapelli RD, Gerschman de Pikelin BS. (1960). Las especies del género *Grammostola* Simon, 1892 en la Republica Argentina.. *Actas Trabajos Congreso Sudamericano de Zoología*.

[bibr60] Schiapelli RD, Gerschman de Pikelin BS. (1970). El género *Ceropelma* Mello-Leitão 1923 (Araneae: Theraphosidae).. *Physis*.

[bibr61] Schiapelli RD, Gerschman de Pikelin BS. (1973). La familia Migidae Simon 1892, en la Argentina (Araneae, Theraphosomorphae).. *Physis*.

[bibr62] Schiapelli RD, Gerschman de Pikelin BS. (1975). *Calathotarsus simoni* sp. nov. (Araneae, Migidae).. *Physis*.

[bibr63] Selléz-Martínez J. (2001). The geology of Ventania (Buenos Aires province, Argentina).. *Journal of Iberian Geology*.

[bibr64] Shillington C, McEwen B. (2006). Activity of juvenile tarantulas in and around the maternal burrow.. *Journal of Arachnology*.

[bibr65] Suero T., Ulibarrena J (1972). Compilación geológica de las Sierras Australes de la provincia de Buenos Aires.. *Ministerio de Obras Publicas* (*La Plata*), *Laboratorio de Ensayo de Materiales*, *Serie II*.

[bibr66] Uetz GW. (1976). Gradient analysis of spider communities in a streamside forest.. *Oecologia*.

[bibr67] Villamil CB, Delucchi G, Long A. (1996). Cincuenta especies prioritarias para su conservación en la provincia de Buenos Aires.. *Resúmenes XXV Jornadas Argentinas de Botánica:*.

[bibr68] Zalba SM, Cozzani NC. (2004). The impact of feral horses on grassland bird communities in Argentina.. *Animal Conservation*.

